# Total Synthesis and Antimicrobial Activity of (±)-Laurelliptinhexadecan-1-one and (±)-Laurelliptinoctadecan-1-one

**DOI:** 10.3390/molecules13122935

**Published:** 2008-11-28

**Authors:** Surachai Nimgirawath, Phansuang Udomputtimekakul, Samathi Pongphuttichai, Asawin Wanbanjob, Thongchai Taechowisan

**Affiliations:** 1 Department of Chemistry, Faculty of Science, Silpakorn University, Nakorn Pathom 73000, Thailand; 2 Department of Microbiology, Faculty of Science, Silpakorn University, Nakorn Pathom 73000, Thailand; E-mail: tthongch@su.ac.th (T. T.)

**Keywords:** Alkaloid, Amidic aporphine, Isoquinoline, Synthesis, Antimicrobial activity

## Abstract

The structures previously assigned to (+)-laurelliptinhexadecan-1-one (**1a**) and (+)-laurelliptinoctadecan-1-one (**1b**) from *Cocculus orbiculatus* (L.) DC. (Menispermaceae) have been confirmed by total synthesis of the racemic alkaloids. The key step of the synthesis involved formation of ring C of the aporphines by a radical-intiated cyclisation. Both (±)-laurelliptinhexadecan-1-one (**1a**) and (±)-laurelliptinoctadecan-1-one (**1b**) were inactive against *Staphylococcus aureus* ATCC25932, *Escherichia coli* ATCC10536 and *Candida albicans* ATCC90028.

## Introduction

**Figure 1:**
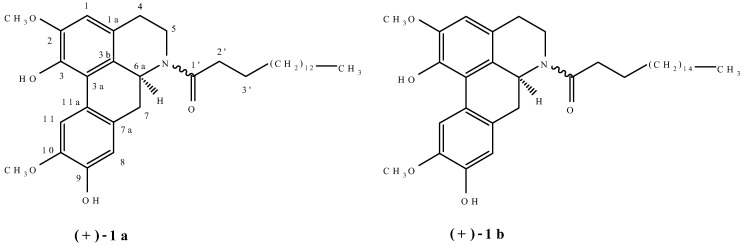
Structures of (+)-laurelliptinhexadecan-1-one (**1**a) and (+)-laurelliptinoctadecan-1-one (**1b**).

Amidic aporphine alkaloids usually occur as *N*-formyl, *N*-acetyl and *N*-methoxycarbonyl derivatives [1]. (+)-Laurelliptinhexadecan-1-one (**1a**) and (+)-laurelliptinoctadecan-1-one (**1b**) are two unique amidic aporphine alkaloids in which a palmitoyl and a stearoyl functional group is attached to the nitrogen of the aporphine nucleus, respectively (Figure 1)[2].

These two unique alkaloids were isolated as an inseparable mixture from *Cocculus orbiculatus* (Menispermaceae). Based on detailed spectroscopic analysis of the mixture, structures **1a** and **1b** were assigned to (+)-laurelliptinhexadecan-1-one and (+)-laurelliptinoctadecan-1-one, respectively. The mixture of **1a** and **1b** was found to exhibit weak activity toward the human hepatoma cell line HepG2 and breast cancer cell line MDA-MB-231. No antimicrobial activity has been reported. In view of the fact that (+)-laurelliptinhexadecan-1-one (**1a**) and (+)-laurelliptinoctadecan-1-one (**1b**) occur as an inseparable mixture, it is therefore desirable to carry out a total synthesis of these two alkaloids to confirm their structures and to study the biological activities of the pure alkaloids.

## Results and Discussion

The syntheses of both (±)-laurelliptinhexadecan-1-one (**1a**) and (±)-laurelliptinoctadecan-1-one (**1b**) were based on the construction of ring C of the aporphine nucleus by a radical-initiated cyclisation [3]. It was initially anticipated that cyclisation of **2b** and **2c** would lead to the desired (±)-**1a** and (±)-**1b** after removal of the benzyl protecting groups. Thus, condensation of 5-benzyloxy-2-bromo-4-methoxyphenylacetyl chloride (**3**) [4] with 4-benzyloxy-3-methoxyphenethylamine (**4**) gave amide **5** which was converted to **6** in a Bischler-Napieralski reaction. Reduction of **6** with sodium borohydride gave (**2a**) which was treated with palmitoyl chloride and stearoyl chloride to give **2b** and **2c** respectively. Unfortunately, several attempts to effect cyclisation of both **2b** and **2c** by a radical-initiated cyclisation were fruitless. Subsequently, isoquinoline **2a** was reacted with trifluoroacetic anhydride to give **2d**, which was treated with tributyltin hydride and azobis(isobutyronitrile) to give **7a** in 22.7% yield and the hydrogenolysis product **2e** in 29.1% yield. Due to steric hindrance, the formation of **7a** was accompanied by concurrent loss of the benzyl protecting group on the oxygen at C-1. Furthermore, the loss of the benzyl radical from the radical intermediate formed after initial cyclisation is also favoured electronically. The structure of **7a** was supported by the presence of a singlet at δ_H_ 8.14 due to the proton at C-11, characteristic of aporphines bearing a proton at that position. Removal of the trifluoroacetyl protecting group was achieved using aqueous potassium carbonate to give **7b** which was treated with palmitoyl chloride and stearoyl chloride to give (±)-9-benzyllaurelliptinhexadecan-1-one (**7c**) and (±)-9-benzyllaurelliptinoctadecan-1- one (**7d**), respectively. Hydrogenolysis of **7c** and **7d** gave (±)-**1a** and (±)-**1b** respectively. Both (±)-**1a** and (±)-**1b** exist as the *Z* and *E* conformers, the ^1^H-NMR spectral data of which are in good agreement with those of natural (+)-**1a** and (+)-**1b** (Table 1 and Table 3).

However, the ^13^C-NMR spectral data of synthetic (±)-**1a** and (±)-**1b** possess a number of signals which are different from those assigned to the same carbons in natural (+)-**1a** and (+)-**1b** (Table 2 and Table 4). These discrepancies may be due to the fact that in the original paper assignments of chemical shifts to the carbon atoms involved were carried out on the spectra measured on the mixture of (+)-**1a** and (+)-**1b**. Bearing this fact in mind, it can be concluded with good confidence that the structures previously assigned to (+)-laurelliptinhexadecan-1-one (**1a**) and (+)-laurelliptinoctadecan-1-one (**1b**) can be confirmed with our current syntheses of the racemic alkaloids. (±)-Laurelliptinhexadecan-1-one (**1a**) and (±)-laurelliptinoctadecan-1-one (**1b**) at the concentration value 256 μg/mL were inactive against *Staphylococcus aureus* ATCC25932, *Escherichia coli* ATCC10536 and *Candida albicans* ATCC90028.

**Scheme 1:**
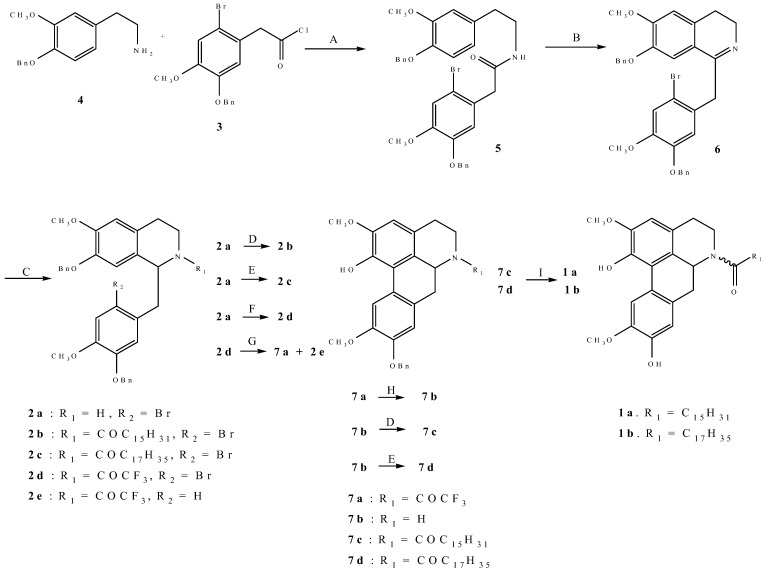
Synthetic routes to (±)-laurelliptinhexadecan-1-one (**1a**) and (±)-laurelliptinoctadecan-1-one (**1b**).

*Reagents and conditions*: A) 10% NaHCO_3_/ chloroform; B) POCl_3_/ benzene; C) NaBH_4_/ethanol; D) palmitoyl chloride, 10 % NaHCO_3_/ chloroform; E) stearoyl chloride, 10 % NaHCO_3_/ chloroform; F) (CF_3_CO)_2_O, Et_3_N/ chloroform; G) Bu_3_SnH, AIBN/ dry toluene; H) K_2_CO_3_/ methanol-water; I) H_2_, Pd/C / ethanol.

**Table 1:** Comparison of ^1^H-NMR spectral data between (+)-laurelliptinhexadecan-1-one (600 MHz, CDCl_3_) and (±)-laurelliptinhexadecan-1-one (300 MHz, CDCl_3_).

	(+)-laurelliptin-hexadecan-1-one *Z*-form	(±)-laurelliptin-hexadecan-1-one *Z*-form	(+)-laurelliptin-hexadecan-1-one *E*-form	(±)-laurelliptin-hexadecan-1-one *E*-form
Position	^1^H	^1^H	^1^H	^1^H
Aporphine moiety				
1	6.55 (s)	6.56 (s)	6.59 (s)	6.60 (s)
1a				
2				
3				
3a				
3b				
4	2.66/2.83 (m)	2.52-2.91 (m)	2.66/2.83 (m)	2.52-2.91 (m)
5	3.23 (pseudo ax., br t, 12.0)	3.24 (pseudo ax., br t, 12.11)	2.75 (pseudo ax., br t, 12.0)	2.75-2.82 (pseudo ax., m)
	4.02 (pseudo eq., br d, 12.0)	4.00 (pseudo eq., br d, 12.11)	4.95 (pseudo eq., br d, 12.0)	4.96 (pseudo eq., br d, 8.03)
6a	5.11 (br d, 12.5)	5.12 (br d, 10.5)	4.60 (br d, 12.5)	4.61 (br d, 12.1)
7	2.61 (pseudo ax., br t, 12.5)	2.58-2.75 (pseudo ax., m)	2.99 (pseudo ax., br t, 12.5)	2.91-3.10 (pseudo ax., m)
	2.95 (pseudo eq., br d, 12.5)	2.91-3.10 (pseudo eq., m)	2.61 (pseudo eq., br d, 12.5)	2.58-2.75 (pseudo eq., m)
7a				
8	6.82 (s)	6.83 (s)	6.82 (s)	6.83 (s)
9				
10				
11	8.06 (s)	8.07 (s)	8.09 (s)	8.10 (s)
11a				
OCH_3_ x 2	ca. 3.90 (s)	3.92 (s)	ca. 3.90 (s)	3.92 (s)
Fatty acid moiety				
1′				
2′	2.44 (m)	2.51-2.40 (m)	2.34 (m)	2.40-2.30 (m)
3′	1.65 (m)	1.78-1.60 (m)	1.65 (m)	1.78-1.60 (m)
Aliphatic CH_2_	1.18-1.30 (m)	1.15-1.40 (m)	1.18-1.30 (m)	1.15-1.40 (m)
Terminal CH_3_	0.86 (t, 6.8)	0.88 (t, 6.8)	0.86 (t, 6.8)	0.88(t, 6.8)

**Table 2:** Comparison of ^13^C-NMR spectral data between (+)-laurelliptinhexadecan-1-one (150 MHz, CDCl_3_) and (±)-laurelliptinhexadecan-1-one (75 MHz, CDCl_3_).

	(+)-laurelliptin-hexadecan-1-one *Z*-form	(±)-laurelliptin-hexadecan-1-one *Z*-form	(+)-laurelliptin-hexadecan-1-one *E*-form	(±)-laurelliptin-hexadecan-1-one *E*-form
position	^13^C	^13^C	^13^C	^13^C
Aporphine moiety				
1	108.3 (d)	108.5 (d)	108.8 (d)	108.9 (d)
**1a**	**111.9 (s)**	**124.2 (s)**	**112.0 (s)**	**125.3 (s)**
2	144.98-145.2 (s)^a^	144.8-145.9 (s)^a^	144.98-145.2 (s)^a^	144.8-145.9 (s)^a^
3	141.0 (s)	141.1 (s)	140.9 (s)	141.1 (s)
**3a**	**125.86 (s)**	**120.6 (s)**	**124.0 (s)**	**120.1 (s)**
**3b**	**120.5 (s)**	**125.6 (s)**	**120.0 (s)**	**126.2 (s)**
**4**	**34.5 (t)**	**30.7 (t)**	**30.6 (t)**	**29.7 (t)**
5	41.4 (t)	41.4 (t)	36.8 (t)	36.7 (t)
6a	50.8 (d)	50.8 (d)	53.4 (d)	53.4 (d)
7	33.2 (t)	33.3 (t)	35.9 (t)	36.1 (t)
7a	123.5 (s)	123.7 (s)	128.8 (s)	123.9 (s)
8	114.5 (d)	114.6 (d)	113.9 (d)	113.9 (d)
9	144.98-145.2 (s)^a^	144.8-145.9 (s)^a^	144.98-145.2 (s)^a^	144.8-145.9 (s)^a^
10	144.98-145.2 (s)^a^	144.8-145.9 (s)^a^	144.98-145.2 (s)^a^	144.8-145.9 (s)^a^
11	**118.8 (d)**	**111.9 (d)**	**111.9 (d)**	**112.0 (d)**
**11a**	**125.4 (s)**	**130.3 (s)**	**125.0 (s)**	**129.6 (s)**
OCH_3_ x 2	56.0 (q) and 56.2 (q)	56.1 (q) and 56.3 (q)	56.0 (q) and 56.2 (q)	56.1 (q) and 56.3 (q)
Fatty acid moiety				
1′	172.1 (s)	171.8 (s)	172.6 (s)	172.5 (s)
2′	33.2 (t)	33.3 (t)	34.2 (t)	34.6 (t)
3′	25.3 (t)	25.3 (t)	25.5 (t)	25.6 (t)
Aliphatic CH_2_	29-32 (t)	29-32 (t)	29-32 (t)	29-32 (t)
Terminal CH_3_	14.1 (q)	14.1 (q)	14.1 (q)	14.1 (q)

^a^ assignments may be interchangeable

**Table 3:** Comparison of ^1^H-NMR spectral data between (+)-laurelliptinoctadecan-1-one (600 MHz, CDCl_3_) and (±)-laurelliptinoctadecan-1-one (300 MHz, CDCl_3_).

	(+)-laurelliptin-octadecan-1-one *Z*-form	(±)-laurelliptin-octadecan-1-one *Z*-form	(+)-laurelliptin-octadecan-1-one *E*-form	(±)-laurelliptin-octadecan-1-one *E*-form
position	^1^H	^1^H	^1^H	^1^H
Aporphine moiety				
1	6.55 (s)	6.53 (s)	6.59 (s)	6.57 (s)
1a				
2				
3				
3a				
3b				
4	2.66/2.83 (m)	2.52-2.88 (m)	2.66/2.83 (m)	2.52-2.88 (m)
5	3.23 (pseudo ax., br t, 12.0)	3.21 (pseudo ax., br t, 12.0)	2.75 (pseudo ax., br t, 12.0)	2.66-2.78 (pseudo ax., m)
	4.02 (pseudo eq., br d, 12.0)	4.00 (pseudo eq., br d, 12.0)	4.95 (pseudo eq., br d, 12.0)	4.95 (pseudo eq., br d, 7.95)
6a	5.11 (br d, 12.5)	5.12 (br d, 13.8)	4.60 (br d, 12.5)	4.61 (br d, 12.1)
7	2.61 (pseudo ax., br t, 12.5)	2.54-2.72 (pseudo ax., m)	2.99 (pseudo ax., br t, 12.5)	2.88-3.50 (pseudo ax., m)
	2.95 (pseudo eq., br d, 12.5)	2.88-3.50 (pseudo eq., m)	2.61 (pseudo eq., br d, 12.5)	2.54-2.72 (pseudo eq., m)
7a				
8	6.82 (s)	6.82 (s)	6.82 (s)	6.82 (s)
9				
10				
11	8.06 (s)	8.06 (s)	8.09 (s)	8.10 (s)
11a				
OCH_3_ x 2	ca. 3.90 (s)	3.89 (s)	ca. 3.90 (s)	3.89 (s)
Fatty acid moiety				
1′				
2′	2.44 (m)	2.50-2.39 (m)	2.34 (m)	2.39-2.29 (m)
3′	1.65 (m)	1.73-1.60 (m)	1.65 (m)	1.73-1.60 (m)
Aliphatic CH_2_	1.18-1.30 (m)	1.15-1.40 (m)	1.18-1.30 (m)	1.15-1.40 (m)
Terminal CH_3_	0.86 (t, 6.8)	0.87 (t, 6.9)	0.86 (t, 6.8)	0.87(t, 6.9)

**Table 4:** Comparison of ^13^C-NMR spectral data between (+)-laurelliptinoctadecan-1-one (150 MHz, CDCl_3_) and (±)-laurelliptinoctadecan-1-one (75 MHz, CDCl_3_).

	(+)-laurelliptin-octadecan-1-one *Z*-form	(±)-laurelliptin-octadecan-1-one *Z*-form	(+)-laurelliptin-octadecan-1-one *E*-form	(±)-laurelliptin-octadecan-1-one *E*-form
position	^13^C	^13^C	^13^C	^13^C
Aporphine moiety				
1	108.3 (d)	108.5 (d)	108.8 (d)	109.0 (d)
**1a**	**111.9 (s)**	**124.2 (s)**	**112.0 (s)**	**125.6 (s)**
2	144.98-145.2 (s)^a^	145.1-145.9 (s)^a^	144.98-145.2 (s)^a^	145.1-145.9 (s)^a^
3	141.0 (s)	141.2 (s)	140.9 (s)	141.1 (s)
**3a**	**125.86 (s)**	**120.7 (s)**	**124.0 (s)**	**120.1 (s)**
**3b**	**120.5 (s)**	**126.0 (s)**	**120.0 (s)**	**125.2 (s)**
**4**	**34.5 (t)**	**30.7 (t)**	**30.6 (t)**	**29.7 (t)**
5	41.4 (t)	41.5 (t)	36.8 (t)	36.8 (t)
6a	50.8 (d)	50.9 (d)	53.4 (d)	53.5 (d)
7	33.2 (t)	33.3 (t)	35.9 (t)	36.1 (t)
7a	123.5 (s)	123.6 (s)	128.8 (s)	123.9 (s)
8	114.5 (d)	114.7 (d)	113.9 (d)	114.1 (d)
9	144.98-145.2 (s)^a^	145.1-145.9 (s)^a^	144.98-145.2 (s)^a^	145.1-145.9 (s)^a^
10	144.98-145.2 (s)^a^	145.1-145.9 (s)^a^	144.98-145.2 (s)^a^	145.1-145.9 (s)^a^
**11**	**118.8 (d)**	**112.1 (d)**	**111.9 (d)**	**112.1 (d)**
**11a**	**125.4 (s)**	**130.1 (s)**	**125.0 (s)**	**129.5 (s)**
OCH_3_ x 2	56.0 (q) and 56.2 (q)	56.1 (q) and 56.3 (q)	56.0 (q) and 56.2 (q)	56.1 (q) and 56.3 (q)
Fatty acid moiety				
1′	172.1 (s)	172.1 (s)	172.6 (s)	172.6 (s)
2′	33.2 (t)	33.3 (t)	34.2 (t)	34.5 (t)
3′	25.3 (t)	25.4 (t)	25.5 (t)	25.5 (t)
Aliphatic CH_2_	29-32 (t)	29-32 (t)	29-32 (t)	29-32 (t)
Terminal CH_3_	14.1 (q)	14.1 (q)	14.1 (q)	14.1 (q)

^a^ assignments may be interchangeable

## Experimental

### General

Melting points were determined on a Stuart Scientific SMP 2 melting point apparatus and are uncorrected. Infrared spectra were recorded on CH_2_Cl_2_-films with a Perkin Elmer Spectrum GX FT-IR spectrophotometer. Ultraviolet spectra were recorded on methanol solutions with a Perkin Elmer Lambda 35 UV-VIS spectrophotometer. ^1^H- and ^13^C-NMR spectra were recorded for deutero- chloroform solutions, unless otherwise stated, at 300 MHz for ^1^H and 75 MHz for ^13^C with a Bruker AVANCE 300 spectrometer. Tetramethylsilane was used as the internal standard. Mass spectra were recorded on a POLARIS Q mass spectrometer. Elemental analysis was performed on a Perkin Elmer 2400 Elemental Analyser.

*2-(2-Bromo-5-benzyloxy-4-methoxyphenyl)-*N*-(4-benzyloxy-3-methoxyphenethyl)acetamide* (**5**)*.* A mixture of 5-benzyloxy-4-methoxy-2-bromophenylacetic acid (26.0 g, 0.07 mol) and thionyl chloride (22.0 g, 0.19 mol) in benzene (150 mL) was refluxed for 1 h. Removal of the solvent under vacuum gave 5-benzyloxy-2-bromo-4-methoxyphenylacetyl chloride (**3**) [4] which was dissolved in ethanol-free chloroform (150 mL) and added to a mixture of 4-benzyloxy-3-methoxyphenethylamine (**4**) (18.1 g, 0.07 mol) in chloroform (150 mL) and 10% sodium hydrogen carbonate (120 mL). The mixture was then stirred for 4 h and the chloroform layer was washed with water (100 mL), 10% hydrochloric acid (100 mL), water (100 mL), then dried over anhydrous sodium sulfate. Removal of the solvent under vacuum gave a residue which was triturated with ethanol to give 2-(2-bromo-5-benzyloxy-4-methoxyphenyl)-*N*-(4-benzyloxy-3-methoxyphenethyl)acetamide (**5**) as white prisms (38.7 g, 93.3%); m.p. 133-134 °C (lit. [5] m.p. 135 °C); ^1^H-NMR: δ 7.45-7.29 (10H, m, Ph-H); 6.99 (1H, s, Ar-H); 6.81 (1H, s, Ar-H); 6.72 (1H, d, *J* = 8.1 Hz, Ar-H); 6.64 (1H, d, *J* = 1.8 Hz, Ar-H); 6.48 (1H, dd, *J* = 8.1, 1.8 Hz, Ar-H); 5.40 (1H, br s, NH); 5.10 (2H, s, CH_2_Ph); 5.08 (2H, s, CH_2_Ph); 3.85 (3H, s, OCH_3_); 3.84 (3H, s, OCH_3_); 3.54 (2H, s, CH_2_CON); 3.42 (2H, apparent q, *J* = 6.6 Hz, CH_2_N); 2.65 (2H, t, *J* = 6.9 Hz, CH_2_); ^13^C-NMR: δ 170.0 (C), 149. (C), 147.8 (C), 146.9 (C), 137.3 (C), 136.4 (C), 131.6 (C), 128.6 (CH), 128.5 (CH), 128.1 (CH), 127.8 (CH), 127.5 (CH), 127.3 (CH), 126.3 (C), 120.6 (CH), 116.4 (CH), 116.1 (CH), 115.4 (C), 114.2 (CH), 112.4 (CH), 109.5 (C), 71.1 (CH_2_), 56.3 (OCH_3_), 56.0 (OCH_3_), 43.5 (CH_2_), 40.7 (CH_2_), 35.0 (CH_2_).

*1-(5-Benzyloxy-2-bromo-4-methoxybenzyl)-7-benzyloxy-6-methoxy-3,4-dihydroisoquinoline* (**6**). Phosphorus oxychloride (75.0 g, 0.49 mol) was added to a solution of 2-(2-bromo-5-benzyloxy-4-methoxyphenyl)-*N*-(4-benzyloxy-3-methoxyphenethyl)acetamide (**5**) (25.0 g, 0.04 mol) in benzene (150 mL) and the solution was refluxed with stirring for 3 h. The reaction mixture was then evaporated under vacuum to yield a brown liquid which was shaken with water (150 mL) and chloroform (150 mL). The mixture was then basified with concentrated ammonia and the chloroform layer was dried over anhydrous sodium sulfate. Removal of the solvent under vacuum gave a dark orange solid which was triturated with ethanol to yield dihydroisoquinoline (**6**) as a pale yellow solid (21.8 g, 90.1%); m.p. 108-110 °C (lit. [6] m.p. 105-106 °C); UV (MeOH) λ_max_ nm (log ε): 207 (4.76), 230 (4.49), 283 (4.00), 311 (3.85); IR ν_max _(film): 3063, 3032, 3005, 2933, 2838, 1621, 1603, 1568, 1506, 1455, 1439, 1378, 1357, 1322, 1256, 1214, 1160, 1142, 1073, 1026, 981, cm^-1^; ^1^H-NMR: δ 7.45-7.20 (10H, m, Ph-H); 7.02 (1H, s, Ar-H); 6.99 (1H, s, Ar-H); 6.78 (1H, s, Ar-H); 6.64 (1H, s, Ar-H); 5.04 (2H, s, CH_2_Ph); 5.03 (2H, s, CH_2_Ph); 4.08 (2H, s, CH_2_); 3.90 (3H, s, OCH_3_); 3.84 (3H, s, OCH_3_); 3.65 (2H, t, *J* = 7.8 Hz, CH_2_N); 2.55 (2H, t, *J* = 7.8 Hz, CH_2_); ^13^C-NMR: δ 167.0 (C), 149.1 (C), 147.7 (C), 146.7 (C), 136.7 (C), 136.6 (C), 132.4 (C), 128.5 (CH), 128.4 (CH), 128.0 (CH), 127.7 (CH), 127.5 (CH), 127.1 (CH), 115.8 (CH), 114.9 (CH), 114.5 (C), 112.9 (C), 112.3 (CH), 110.7 (CH), 106.0 (C), 105.4 (C), 71.2 (CH_2_), 70.9 (CH_2_), 56.2 (OCH_3_), 56.1 (OCH_3_), 46.1 (CH_2_), 41.2 (CH_2_), 25.7 (CH_2_).

*1-(5-Benzyloxy-2-bromo-4-methoxybenzyl)-7-benzyloxy-6-methoxy-1,2,3,4-tetrahydroisoquinoline* (**2a**). Sodium borohydride (11.1 g, 0.29 mol) was added portionwise to a stirred solution of dihydroisoquinoline (**6**) (15.0 g, 0.03 mol) in ethanol (150 mL) and the mixture was stirred for 3 h. Removal of the ethanol under vacuum gave a residue which was shaken with water (100 mL) and chloroform (100 mL) and the chloroform layer was dried. Removal of the solvent gave a crude white solid which was triturated with ethanol to give tetrahydroisoquinoline **2a** as a yellow solid (14.0 g, 93.0%); m.p. 91-93 °C (Lit. [7] m.p. 90-90.5 °C); UV (MeOH) λ_max_ nm (log ε): 211 (4.48), 236sh (4.26), 290sh (3.79), 324 (3.70); IR ν_max_ (film): 3585, 3063, 3032, 3007, 2936, 2839, 1592, 1567, 1506, 1456, 1438, 1410, 1383, 1356, 1334, 1320, 1267, 1217, 1173, 1143, 1081, 1053, 1024, 958 cm^-1^; ^1^H-NMR: δ 7.46-7.20 (10H, m, Ph-H); 7.06 (1H, s, Ar-H); 6.74 (1H, s, Ar-H); 6.71(1H, s, Ar-H); 6.59 (1H, s, Ar-H); 5.11(4H, s, 2 x CH_2_Ph); 4.05-3.97 (1H, m, H-1); 3.85 (6H, s, 2 x OCH_3_); 3.13-3.01 (2H, m, CH_2_); 2.87-2.64 (4H, m, 2 x CH_2_); ^13^C-NMR: δ 149.1 (C), 148.3 (C), 147.1 (C), 146.2 (C), 137.4 (C), 136.7 (C), 130.5 (C), 130.3 (C), 128.6 (CH), 128.0 (CH), 127.9 (C), 127.8 (CH), 127.4 (CH), 127.3 (CH), 117.3 (CH), 116.3 (CH), 115.4 (C), 112.9 (CH), 112.2 (CH), 71.4 (CH_2_), 71.1 (CH_2_), 56.3 (OCH_3_), 56.0 (OCH_3_), 55.1 (CH), 42.3 (CH_2_), 40.1 (CH_2_), 29.4 (CH_2_).

*1-(5-Benzyloxy-2-bromo-4-methoxybenzyl)-7-benzyloxy-6-methoxy-2-trifluoroacetyl-1,2,3,4-tetra-hydroisoquinoline* (**2d**). Trifluoroacetic anhydride (54.0 g, 0.26 mol) was added to a stirred solution of tetrahydroisoquinoline **2a** (23.2 g, 0.04 mol) and triethylamine (36.0 g) in chloroform (300 mL) at 0-10 °C. Stirring was continued at room temperature for 3 h. Chloroform (200 mL) was added and the chloroform layer was washed with 10% NaHCO_3_ (4 x 300 mL), water (300 mL), 10% HCl (6 x 300 mL) and water (300 mL), then dried. Removal of the solvent under vacuum gave a brown solid which was triturated with ethanol to yield trifluoroacetyltetrahydroisoquinoline **2d** as a pale yellow solid (17.3 g, 63.9%); m.p. 157-158 °C; UV (MeOH) λ_max_ nm (log ε): 208 (4.75), 232sh (4.12), 287 (3.65); IR ν_max _(film): 3033, 2919, 2849, 1689, 1609, 1509, 1456, 1440, 1381, 1258, 1197, 1165, 1141, 1116, 1027, 912 cm^-1^; Anal. Calc. for C_34_H_31_NO_5_BrF_3_: C 60.90, H 4.66, N 2.09%. Found: C 60.72, H 4.81, N 2.25%. ^1^H-NMR: δ 7.45-7.30 (10H, m, Ph-H); 7.01 (1H, s, Ar-H); 6.61 (1H, s, Ar-H); 6.60 (1H, s, Ar-H); 6.45 (1H, s, Ar-H); 5.65-5.57 (1H, m, H-1); 5.00 (2H, s, CH_2_Ph); 4.98 (2H, s, CH_2_Ph); 4.02-3.91 (1H, m, H-3α); 3.88 (3H, s, OCH_3_); 3.85 (3H, s, OCH_3_); 3.64-3.53 (1H, m, H-3β); 3.28-2.68 (4H, m, 2 x CH_2_); ^13^C-NMR: δ (both conformers) 155.8 (C), 149.3 (C), 148.9 (C), 147.4 (C), 146.8 (C), 136.9(C), 136.7 (C), 128.7 (CH), 128.6 (CH), 128.3 (C), 128.1 (CH), 128.0 (CH), 127.4 (CH), 127.3 (CH), 127.2 (CH), 126.4 (C), 125.5 (C), 118.4 (C), 116.7 (CH), 116.5 (CH), 116.0 (C), 115.8 (CH), 114.6 (C), 112.9 (CH), 112.7 (CH), 111.8 (CH), 111.4 (CH), 71.4 (CH_2_), 71.3 (CH_2_), 71.2 (CH_2_), 56.6 (OCH_3_), 56.2 (OCH_3_), 56.1 (OCH_3_), 56.0 (OCH_3_), 54.1 (CH), 42.3 (CH_2_), 41.8 (CH_2_), 40.8 (CH_2_), 40.2 (CH_2_), 40.1 (CH_2_), 37.5 (CH_2_), 28.7 (CH_2_), 27.2 (CH_2_).

*9-Benzyloxy-2,10-dimethoxy-1-hydroxy-6-trifluoroacetylnoraporphine* (**7a**). A solution of azobis(isobutyronitrile) (3.7 g, 0.02 mol) and tributyltin hydride (25.2 g, 0.09 mol) in toluene (160 mL) was added dropwise in four equal portions over 3 h to a refluxing solution of trifluoroacetyltetrahydroisoquinoline **2d** (16.0 g, 0.02 mol) in toluene (250 mL) and the resulting mixture was then refluxed for another 24 h. The solvent was then removed under vacuum and the residue was dissolved in acetonitrile (200 mL) and washed with hexane (3 x 200 mL), then dried. Removal of the solvent gave a brown solid which was triturated with ethanol to give crude noraporphine **7a** (6.92 g), which was separated on a silica gel column using benzene-chloroform as eluent. The earlier fractions gave the hydrogenolysis product **2e** as a white solid (4.1 g, 29.1%); m.p. 85-86 °C; ^1^H-NMR: δ 7.48-7.25 (10H, m, Ph-H of both conformers); 6.79 and 6.74 (total 1H, 2 d, *J* = 8.6 and 8.2 Hz, Ar-H of both conformers); 6.61-6.55 (total 2H, m, Ar-H of both conformers); 6.53-6.46 (total 1H, m, Ar-H of both conformers); 6.27 and 6.25 (total 1H, 2 s, Ar-H of both conformers); 5.44 (1H, apparent t, *J* = 6.2 Hz, H-6a of both conformers); 5.17, 4.90 (total 2H, 2 AB q, *J* = 12.2 and 12.4 Hz, CH_2_Ph of both conformers); 5.08, 4.98 (total 2H, 2 s, CH_2_Ph of both conformers); 3.93, 3.90, 3.84 and 3.82 (total 6H, 4 s, 2 x OCH_3_ of both conformers); 3.33-3.20 (1H, m, CH_2_ of both conformers); 3.00-2.50 (5H, m, CH_2_ of both conformers);^ 13^C-NMR: δ (both conformers) 155.8 (C), 148.9 (C), 148.7 (C), 148.0 (C), 146.5 (C), 137.0 (C), 136.9 (C), 129.3 (C), 128.6 (CH), 128.5 (CH), 127.9 (CH), 127.8 (CH), 127.4 (CH), 127.2 (CH), 126.4 (C), 125.7 (C), 122.6 (CH), 118.5 (C), 115.4 (CH), 113.1 (CH), 111.6 (CH), 111.5 (CH), 70.9 (CH_2_), 70.9 (CH_2_), 56.3 (OCH_3_), 56.2 (OCH_3_), 56.0 (OCH_3_), 55.2 (CH), 41.0 (CH_2_), 40.6 (CH_2_), 28.5 (CH_2_). The later fractions gave pure noraporphine **7a** as a yellow-white solid (2.7 g, 22.7%); m.p. 232-234 °C; UV (MeOH) λ_max_ nm (log ε): 213 (4.57), 272sh (3.99), 281 (4.08), 306 (4.12); IR ν_max _(film): 3373, 2920, 2850, 1682, 1605, 1513, 1463, 1401, 1383, 1371, 1337, 1310, 1281, 1255, 1202, 1169, 1139, 1099, 1049, 1025, 928 cm^-1^; Anal. Calc. for C_27_H_24_NO_5_F_3_: C 64.93, H 4.84, N 2.80%. Found: C 65.09, H 4.68, N 2.65%. ^1^H-NMR: δ 8.14 (1H, s, H-11), 7.60-7.30 (5H, m, Ph-H); 6.80 (1H, s, H-8), 6.57 (1H, s, H-3), 6.23 (1H, s, OH), 5.18 (2H, AB q, *J* = 12.3 Hz, CH_2_Ph), 5.09-5.00 (1H, m, H-6a), 4.25-4.14 (1H, m, H-5α), 3.94 (3H, s, OCH_3_), 3.93 (3H, s, OCH_3_), 3.38-3.26 (1H, m, H-5β), 3.04-2.66 (4H, m, 2 x CH_2_); ^13^C-NMR: δ 155.9 (C), 148.0 (C), 147.5 (C), 146.0 (C), 141.6 (C), 137.1 (C), 128.6 (CH), 128.3 (C), 127.9 (CH), 127.3 (CH), 124.6 (C), 124.1 (C), 123.7 (C), 120.2 (C), 118.3 (C), 113.8 (CH), 113.1 (CH), 108.7 (CH), 70.9 (CH_2_), 56.3 (OCH_3_), 56.2 (OCH_3_), 52.6 (CH), 41.4 (CH_2_), 32.9 (CH_2_), 30.2 (CH_2_).

*9-Benzyloxy-2,10-dimethoxy-1-hydroxynoraporphine (***7b**). A mixture of the noraporphine **7a** (1.6 g, 3.21 mmol), potassium carbonate (3.0 g), methanol (150 mL) and water (9 mL) was refluxed for 4 h. The solvent was then removed under vacuum and 5% sodium bicarbonate (100 mL) was added to the residue, which was extracted with chloroform (3 x 30 mL), then dried over anhydrous sodium sulfate. Removal of the solvent gave a dark-brown solid which was triturated with ethanol to give noraporphine **7b** as a brown solid (996.7 mg, 77.3%); m.p. 197-198 °C; UV (MeOH) λ_max_ nm (log ε): 217 (4.62), 270sh (4.12), 280 (4.22), 305 (4.23); IR ν_max _(film): 3583, 3292, 2919, 2849, 1603, 1509, 1463, 1455, 1398, 1373, 1284, 1252, 1213, 1124, 1102, 1038, 1025, 992 cm^-1^; MS (EI) m/z (%): 403 (M^+^, 32), 312 (100), 91 (7); Anal. Calc. for C_25_H_25_NO_4_: C 74.42, H 6.24, N 3.47%. Found: C 74.26, H 6.38, N 3.66%. ^1^H-NMR: δ 8.09 (1H, s, H-11); 7.50-7.28 (5H, m, Ph-H); 6.76 (1H, s, H-8); 6.54 (1H, s, H-3); 5.17 (2H, s, CH_2_Ph); 3.91 (3H, s, OCH_3_); 3.89 (3H, s, OCH_3_); 3.40-3.31 (1H, m, H-5α); 3.05-2.58 (6H, m, H-5β, CH, 2 x CH_2_); ^13^C-NMR: δ 147.9 (C), 146.9 (C), 145.9 (C), 140.8 (C), 137.3 (C), 128.7 (C), 128.5 (CH), 128.4 (C), 127.8 (CH), 127.3 (CH), 125.5 (C), 124.1 (C), 119.1 (C), 113.6 (CH), 113.0 (CH), 109.4 (CH), 71.0 (CH_2_), 56.2 (OCH_3_), 56.1 (OCH_3_), 53.8 (CH), 43.3 (CH_2_), 36.9 (CH_2_), 29.0 (CH_2_).

*9-Benzyloxy-2,10-dimethoxy-1-hydroxy-6-palmitoylnoraporphine* (**7c**). A solution of palmitoyl chloride (389.0 mg, 1.42 mmol) in chloroform (80 mL) was added to a stirred mixture of noraporphine **7b** (400.0 mg, 0.99 mmol) in chloroform (80 mL) and 10% sodium bicarbonate (120 mL). Stirring was continued overnight. The chloroform layer was separated, then dried. Removal of the solvent gave a brown solid which was recrystallized with ethanol to give palmitoylnoraporphine **7c** as a yellow-white solid (530.0 mg, 83.3%); m.p. 85-86°C; UV (MeOH) λ_max_ nm (log ε): 218 (4.45), 273sh (3.98), 281(4.04), 306 (4.10), 314sh (4.07); IR ν_max_ (film): 3503, 2923, 2852, 1635, 1605, 1515, 1463, 1456, 1428, 1398, 1338, 1277, 1249, 1215, 1192, 1165, 1123, 1098, 1027 cm^-1^; MS (EI) m/z (%): 641(M^+^, 63), 550(10), 386(100), 295(79), 91 (15); Anal. Calc. for C_41_H_55_NO_5_: C 76.72, H 8.64, N 2.18%. Found: C 76.62, H 8.80, N 2.35%. ^1^H-NMR: δ (both conformers) 8.15, 8.13 (total 1H, 2 s, H-11); 7.50-7.28 (total 5H, m, Ph-H); 6.81, 6.79 (total 1H, 2 s, H-8); 6.59, 6.55 (total 1H, 2 s, H-3); 6.23 (1H, br s, OH); 5.26-5.15 (total 2H, m, CH_2_Ph); 5.13, 4.60 (total 1H, 2 br d, *J* = 3.8 and 13.2 Hz, H-6a); 4.94, 4.01 (total 1H, 2 br d, *J* = 8.1 and 10.1 Hz, H-5); 3.92 (3H, s, OCH_3_); 3.89 (3H, s, OCH_3_); 3.21 and 2.78-2.68 (total 1H, br t, *J* = 12.1 Hz, and m, H-5); 3.03-2.87 and 2.74-2.54 (total 2H, m, CH_2_-7); 2.97-2.59 (total 2H, m, CH_2_-4); 2.43-2.31 ( total 2H, m, CH_2_-2′); 1.75-1.55 (2H, m, CH_2_-3′); 1.45-1.15 (24H, m, aliphatic CH_2_); 0.88 (3H, t, *J* = 6.6 Hz, terminal CH_3_); ^13^C-NMR: δ (both conformers) 172.6 (C), 172.0 (C), 148.2 (C), 147.7 (C), 147.3 (C), 145.9 (C), 145.7 (C), 141.4 (C), 141.3 (C), 137.2 (C), 137.0 (C), 129.5 (C), 128.5 (CH), 127.8 (CH), 127.3 (CH), 126.1 (C), 125.6 (C), 125.1 (C), 124.8 (C), 124.2 (C), 120.4 (C), 119.9 (C), 113.8 (CH), 113.3 (CH), 113.1 (CH), 109.1 (CH), 108.7 (CH), 71.1 (CH_2_), 70.8 (CH_2_), 56.3 (OCH_3_), 56.2 (OCH_3_), 53.4 (CH), 50.9 (OCH_3_), 41.4 (CH_2_), 36.8 (CH_2_), 36.2 (CH_2_), 34.5 (CH_2_), 34.0 (CH_2_), 33.4 (CH_2_), 31.9 (CH_2_), 30.7 (CH_2_), 29.7 (CH_2_), 29.5 (CH_2_), 29.4 (CH_2_), 29.3 (CH_2_), 29.2 (CH_2_), 25.6 (CH_2_), 25.3 (CH_2_), 24.9 (CH_2_), 22.7 (CH_2_), 14.1 (CH_3_).

*9-Benzyloxy-2,10-dimethoxy-1-hydroxy-6-stearoylnoraporphine* (**7d**). In a similar manner, stearoylnoraporphine **7d** was obtained in 88.4% yield as a yellow-white solid from ethanol; m.p. 83-84 °C; UV (MeOH) λ_max_ nm (log ε): 218 (4.37), 272sh (3.69), 281 (3.79), 305 (3.87), 314sh (3.82); IR ν_max_ (film): 3393, 2923, 2852, 1641, 1603, 1513, 1464, 1428, 1399, 1337, 1255, 1214, 1115, 1100, 1026 cm^-1^; MS (EI) m/z (%): 669 (M^+^, 6), 578 (1), 386 (10), 296 (100), 91 (3); Anal. Calc. for C_43_H_59_NO_5_: C 77.09, H 8.88, N 2.09%. Found: C 77.26, H 8.65, N 2.23%. ^1^H-NMR: δ (both conformers) 8.15, 8.13 (total 1H, 2 s, H-11); 7.50- 7.28 (total 5H, m, Ph-H); 6.82, 6.79 (total 1H, 2 s, H-8); 6.60, 6.56 (total 1H, 2 s, H-3); 6.19 (1H, br s, OH); 5.20-5.11 (total 2H, m, CH_2_Ph); 5.10, 4.60 (total 1H, 2 br d, *J* = 9.7 and 12.6 Hz, H-6a); 4.95, 4.03 (total 1H, 2 br d, *J* = 7.7 and 13.6 Hz, H-5); 3.92 (3H, s, OCH_3_); 3.89 (3H, s, OCH_3_); 3.22 and 2.76-2.68 (total 1H, br t, *J* = 12.1 Hz and m, H-5); 3.07-2.89 and 2.71-2.55 (total 2H, m, CH_2_-7); 2.90-2.55 (total 2H, m, CH_2_-4); 2.43-2.33 (total 2H, m, CH_2_-2′); 1.75-1.55 (2H, m, CH_2_-3′); 1.40-1.15 (28H, m, aliphatic CH_2_); 0.88(3H, t, *J* = 6.7 Hz, terminal CH_3_); ^13^C-NMR: δ (both conformers) 172.5 (C), 171.9 (C), 148.3 (C), 147.8 (C), 147.3 (C), 145.9 (C), 145.7 (C), 141.4 (C), 137.2 (C), 137.1 (C), 129.5 (C), 128.5 (CH), 127.8 (CH), 127.3 (CH), 126.2 (C), 125.6 (C), 125.2 (C), 124.8 (C), 124.2 (C), 120.4 (C), 120.0 (C), 113.8 (CH), 113.3 (CH), 113.1 (CH), 109.1 (CH), 108.7 (CH), 71.1 (CH_2_), 70.9 (CH_2_), 56.3 (OCH_3_), 56.2 (OCH_3_), 53.4 (CH), 50.9 (CH), 41.4 (CH_2_), 36.8 (CH_2_), 36.3 (CH_2_), 34.5 (CH_2_), 33.4 (CH_2_), 31.9 (CH_2_), 30.7 (CH_2_), 29.7 (CH_2_), 29.5 (CH_2_), 29.4 (CH_2_), 25.6 (CH_2_), 25.3 (CH_2_), 22.7 (CH_2_), 14.1 (CH_3_).

*(±)-Laurelliptinhexadecan-1-one* (**1a**). A solution of noraporphine **7c** (300.5 mg, 0.47 mmol) in ethanol (70 mL), was hydrogenolysed over Pd/C (31.1 mg) at atmospheric pressure for 48 h. The catalyst was filtered off and the solvent removed under vacuum. The resulting white residue was recrystallized from ethanol to give (±)-laurelliptinhexadecan-1-one (**1a**) as a gray-white solid (182.0 mg, 70.5%); m.p. 147-148 °C; UV (MeOH) λ_max_ nm (log ε): 221 (4.57), 272sh (4.13), 282 (4.20), 305 (4.27), 315sh (4.24); IR ν_max_ (film): 3370, 2923, 2852, 1622, 1602, 1513, 1464, 1431, 1413, 1366, 1330, 1279, 1246, 1193, 1122, 1096, 1035, 960 cm^-1^; MS (EI) m/z (%): 551 (M^+^, 26), 296 (100); Anal. Calc. for C_34_H_49_NO_5_: C 74.01, H 8.95, N 2.54%. Found: C 74.19, H 9.07, N 2.68%. ^1^H-NMR and ^13^C-NMR see Table 1 and Table 2.

*(±)-Laurelliptinoctadecan-1-one* (**1b**). In a similar manner, (±)-laurelliptinoctadecan-1-one (**1b**) was obtained in 79.2% yield as a gray-white solid from ethanol; m.p. 146-147 °C; UV (MeOH) λ_max_ nm (log ε): 221 (4.61), 271sh (4.11), 282 (4.20), 305 (4.28), 313sh (4.25); IR ν_max_ (film): 3383, 2923, 2852, 1625, 1600, 1509, 1463, 1413, 1366, 1330, 1279, 1246, 1193, 1122, 1096, 1034, 960 cm^-1^; MS (EI) m/z (%): 579 (M^+^, 17), 296 (100); Anal. Calc. for C_36_H_53_NO_5_: C 74.57, H 9.21, N 2.42%. Found: C 74.7, H 9.12, N 2.36%.^1^H-NMR and ^13^C-NMR see Table 3 and Table 4.

### Minimum inhibitory concentration (MIC)

The MICs of (±)-laurelliptinhexadecan-1-one and (±)-laurelliptinoctadecan-1-one were determined by the NCCLS microbroth dilution method [8]. (±)-Laurelliptinhexadecan-1-one and (±)-laurelliptinoctadecan-1-one were weighed and dissolved in DMSO to make a solution of concentration 2.56 mg/mL. From this stock solution two-fold serial dilution was carried out with culture medium in 96-well microplates (100 μl of total volume) to give a series of solutions ranging from 256 μg/mL to 0.50 μg/mL. Three different microorganisms were selected *viz. Staphytolcoccus aureus* ATCC25932, *Escherichia coli* ATCC10536 and *Candida albicans* ATCC90028. They were subcultured on nutrient broth supplemented with 10% glucose (NBG) (for bacteria) or Sabouraud glucose broth (for yeast) and incubated at 37 ^o^C for 24 h. A final concentration of 1 x 10^5^ cfu/mL of test bacteria or yeast was added to each dilution. The plates were incubated at 37 ^o^C for 48 h. MIC was defined as the lowest concentration of test agent that inhibited bacterial or yeast growth, as indicated by the absence of turbidity. Test agent-free broth containing 5% DMSO was incubated as growth control.
